# Supplementary material for: An enhancer RNA recruits MLL1 to regulate transcription of Myb
Source: bioRxiv. 2023 Oct 12:2023.09.26.559528. Originally published 2023 Sep 26. Preprint. [Version 2] doi: 10.1101/2023.09.26.559528 (PMC10557664; doi:10.1101/2023.09.26.559528)
Supplement: Supplement 1 [file media-1.pdf]

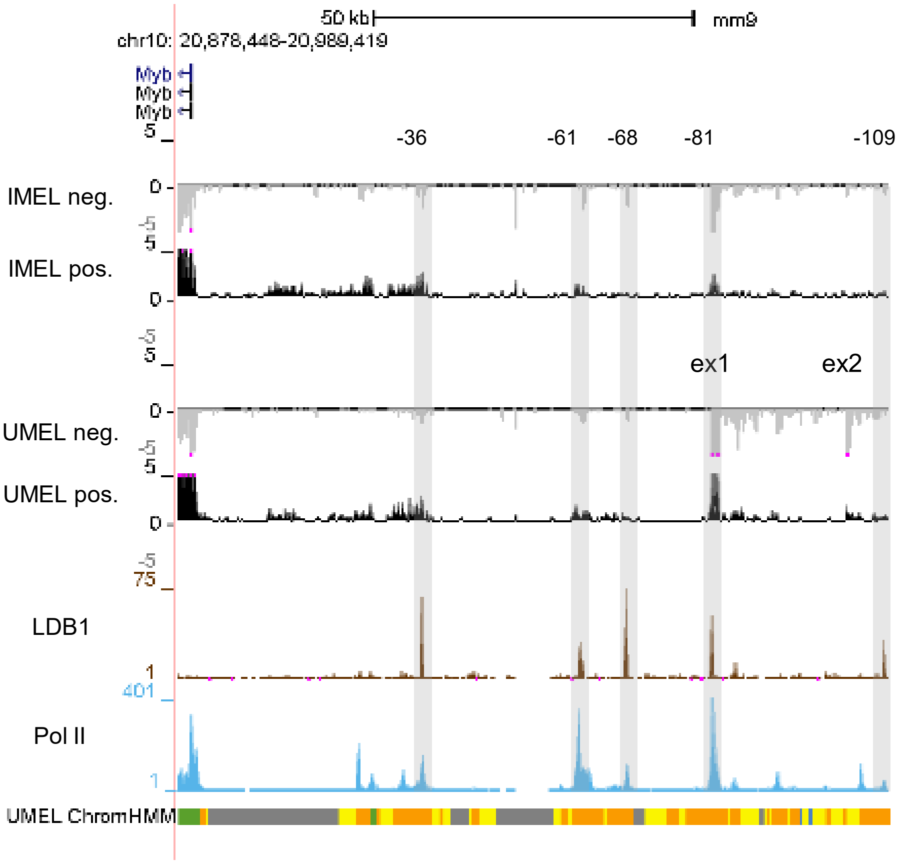

Figure S1. RNA-seq results for the *Myb*-Hbsl1 intergenic region. Signal tracks for RNA-seq of induced (IMEL) and uninduced (UMEL) MEL cells from ENCODE are shown together with signal tracks for LDB1 and Pol II. Related to Figure 1.

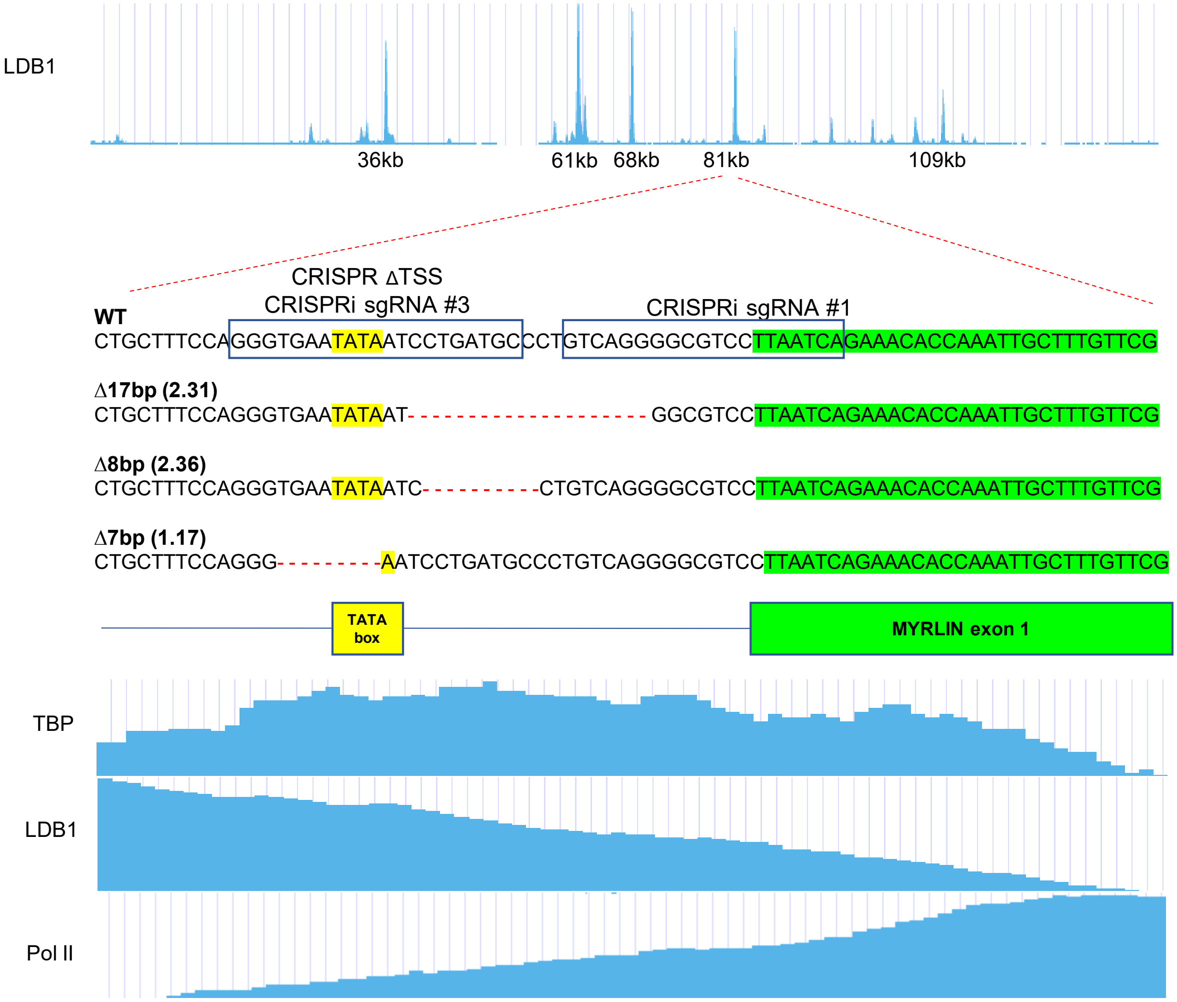

Figure S2. Schematic of the *Myb-Hbsl1* intergenic region. The zoomed in portion at the -81kb enhancer shows the gRNA used for targeting the *Myrlin* TSS, the resulting three CRISPR-Cas9 deletions from Figure 2, the TATA box and the transcription start site for *Myrlin*. Below are signal tracks for TBP, LDB1 and Pol II from ENCODE. The locations of gRNAs used for CRISPR targeting of dCAS9-KRAB are also indicated. Related to Figure 2.

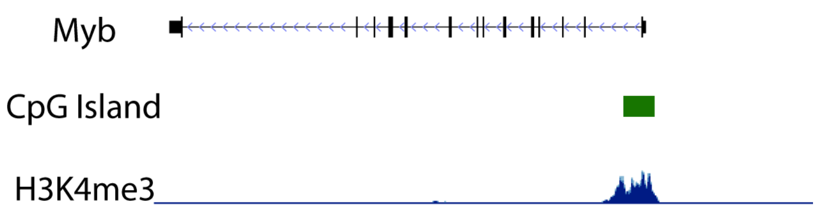

Figure S3. Encode genome browser view of the CpG island in the *Myb* promoter. The *Myb* promoter and first exon/intron contain a CpG island, which is highly enriched for H3K4me3 when *Myb* is actively transcribed. Related to Figure 5.

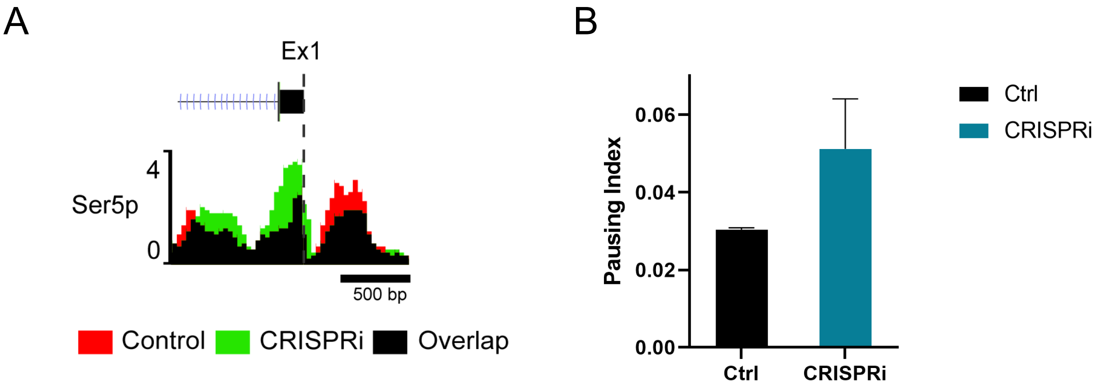

Figure S4. **Pol II Ser5P occupancy in the *Myb* promoter.** (A) Analysis of the Pol II Ser5P CUT & TAG fragments displaying Pol II Ser5 occupancy. (B) Pausing index calculated for Pol II Ser5 across *Myb*. Error bars indicate SEM of 2 independent experiments. Related to Figure 6.

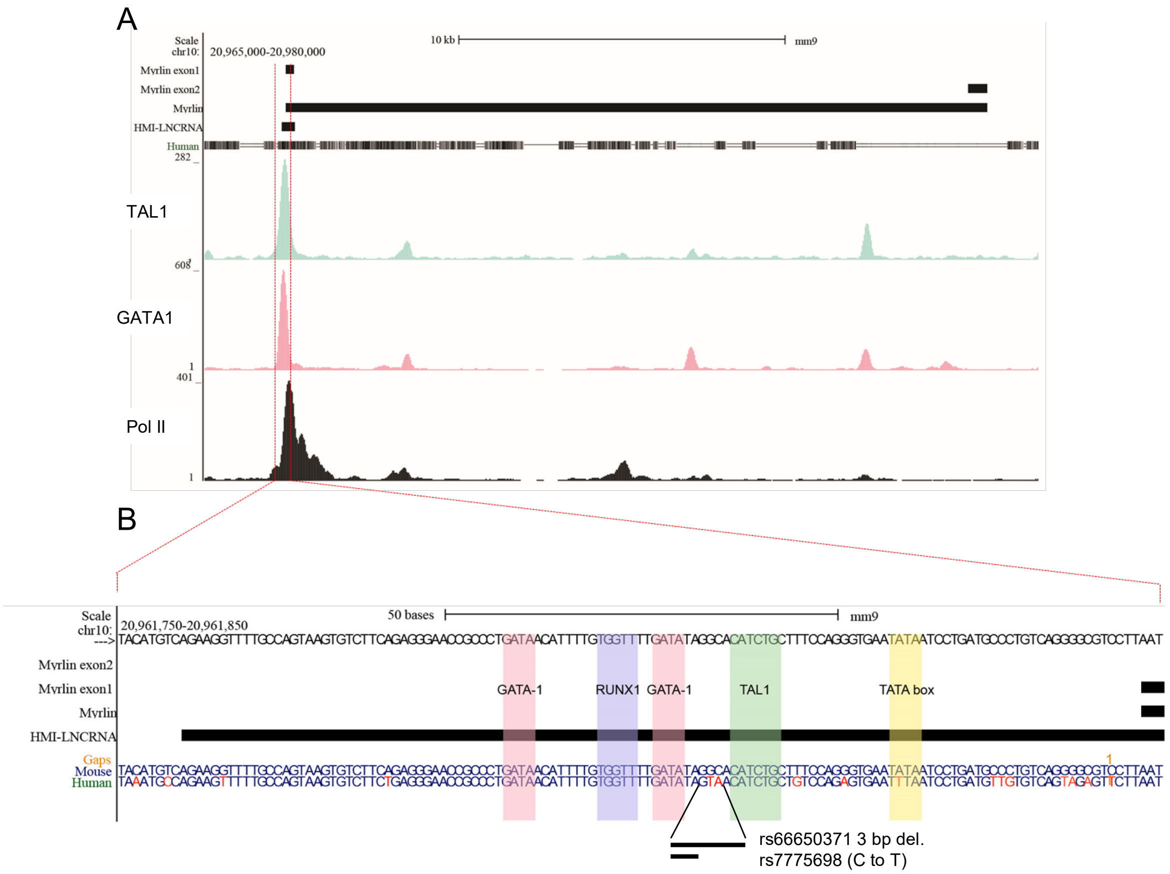

Figure S5. **Human and mouse *Myb* enhancer transcripts.** (A) Illustration of genomic region between chr10: 20,965,000 – 20,980,000 (mm9), showing BLATN result for human HMI-LNCRNA lncRNA and a 378 bp homology patch (see text) shared with the mouse *Myrlin* locus. Below are ENCODE ChIP-seq signal tracks for TAL1, GATA1, and Pol2 at the -81kb intergenic enhancer. (B) Chr10: 20,961,750 – 20,961,850 (mm9) zoom-in snapshot (dashed red lines) including HMI-LNCRNA DNA homology patch and highlighting GATA1, RUNX1, and TAL1 motifs conserved between mouse and human. The TATA box motif (yellow) appears only in the mouse reference genome. Below are the mouse and human DNA sequence showing nucleotide differences (red) with human single nucleotide polymorphisms (SNPs) rs66650371 and rs7775698 that are among the SNPs in the MYB locus that are linked to elevated fetal hemoglobin in humans.

Table S1. List of primers and oligos. Related to STAR Methods.

	Sequence 5'-3'
CRISPR-gRNA	
gRNA1 -81kb TSS SE	CACCGTCAGGGGCGTCCTTAATCA
gRNA1 -81kb TSS AE	AAACTGATTAAGGACGCCCCTGAC
gRNA2 -81kb TSS SE	CACCGGACGCCCCTGACAGGGCATC
gRNA2 -81kb TSS AE	AAACGATGCCCTGTCAGGGGCGTCC
gRNA3 -81kb TSS SE	CACCGCATCAGGATTATATTCACCC
gRNA3 -81kb TSS AE	AAACGGGTGAATATAATCCTGATGC
RACE	
3' RACE outer	GAGTTTCACAACCACCTTCCAA
3' RACE inner	GAGAACAACAGTGGATGGGC
5' RACE outer	TAGCTGAGGGAACAAACGGAG
5' RACE inner	CTCTGGCATAACTGGAGTAAATTA
PCR	
Myrlin exon 1 F	TTAATCAGAAACACCAAATTGCTT
Myrlin exon 1 F	CTGTAGCTGAGGGAACAAACG
Myrlin exon 2 F	ATCAGGATACAGCTCTCAGCTACT
Myrlin exon 2 R	CGAGGCAATCTCTTGGTTTTA
RT & ChIP qPCR	
Actin exon F	ACCTTCTACAATGAGCTGCG
Actin exon R	CTGGATGGCTACGTACATGG
Actin intron F	GGGGCTCCACTTAGACCTAC
Actin intron R	GGCTCGTGTGACAAAGCTAA
Myrlin exon 1 F	AAATTGCTTTGTTCGCCTGC
Myrlin exon 1 R	CGAGTACCGCTTTCACAGTT
Myrlin exon 1/2 junction F	GTTCCGGCTCCGTTTGTT
Myrlin exon 1/2 junction R	CATCATTATGGTGGGGAACAT
Myrlin exon 2 F	TCAGCTACTGTTCCAGTGCC
Myrlin exon 2 R	GGGCTTACAGTTCCAGAGGA
Malat1 F	CAGGAGTGAGGCTTGTGGTA
Malat1 R	TGAATGGTAGAGCCAGCACA
Myb prom F	TCTTTGTTTGATGGCATCTGTT
Myb prom R	AAAGGGGAGGAGAAGGAGGT
-36kb F	TCACTTCCTTCCTGTCTCG
-36kb R	GTCTGGTGGCGATGACTTA
-61kb F	GTTGGGCAAAGATACTGGAT
-61kb R	GTTGGGCAAAGATACTGGAT
-68kb F	ATCCAAGTGACGGTGACA
-68kb R	GCATCCTGATTGTGCTAACT
-81kb F	CCCAAGTGAGAGAAATGTTGAA
-81kb R	TGTTATCAGGGCGGTTCC

-109kb F	CTCCAACATCAGCCGACT
-109kb R	ATTGTGAGGTGAGTGCCC
Myb exon 2 F	TTTCCAGATTTGGGCAGCAG
Myb exon 2 R	TGCAGCATCTACAGTAGCGA
Myb exon 3 F	GGTCCAGGGACCTTTGATGA
Myb exon 3 R	GACAGATGTGCAGTGCCAA
Necdin F	AGCTCATGTGGTACGTGTTGGT
Necdin R	GCTGCCCATGACCTCTTTCA
HS2 F	ATGTTAGTGTCAGCATATTACCGATGT
HS2 R	AATGCAGAGGCTCGTTAATGTG
Myb int1 F	GAACTGCAAACCAGCCTCTT
Myb int1 R	GGGATGTTTCATCCTGAGAGC
Hbb-bh1 F	CCTGGCCATCATGGGAAAC
Hbb-bh1 R	CCCCAAGCCCAAGGATGT
Hbb-b1 F	CACATGCAGCTTGTCACAGT
Hbb-b1 R	GTCAAAGCCCATGGCAAGAA
Hbb-y F	GCTTGTCACAGTGCAGTTCA
Hbb-y R	CGATGGCCTGAATCACTTGG
Chromatin Capture 3C Taqman qPCR	
Myb prom universal F	AGTTCAAGACTTGTGCTGACTG
Myb prom universal Probe	5’-FAM-AACCCCTAAAGCACTTGGGACACTCACAC-3’-BHQ1
20898686 R	CCTAGGTTCTCCTCTCCTACCA
20904395 R	GACAATTTGACATGAATTGCAAGC
20909424 R	CAAGAACCAAGACGCCTCAG
20915439 R	AGTAAATCTTGCTGCCCTCAAG
20923231 R	TGCCTTGGGCAGTTCTATAGAG
20939397 R	TCCTTACTTCTGCTCTCAAACAC
20944478 R	CTTTGTAGGTCACTTTCTCCAGC
20947418 R	AGCAGGCTATTGTGAAAAGAGG
20950525 R	ACACATAGGCACAGAGGAAAGTT
20957119 R	GCAACCTTTTCACTGGCAGAAAT
20974886 R	GAAGACCACTTAGGTAAACACTTTG
20978637 R	TGACACATTTGCTGCGAACAG
20986398 R	TGGAAAAGATCAGTAAGGCCAGA
21006046 R	ACTACATAACTTGGGTGTGTTGG
21013311 R	TGCTTCTTGGTCCCGGTGT
ChIRP probe	
Probe 1	CAGTGTTGCAGGCGAACAAA- Bio-TEG
Probe 2	TTGTTCTCTGGCATAACTGG- Bio-TEG
Probe 3	CACTGGAACAGTAGCTGAGA- Bio-TEG
Probe 4	AGACACCATGACCAAGACGA- Bio-TEG
Probe 5	CCAAAATCCACATTCTGTCA- Bio-TEG
Probe 6	AACTCCTTTCAGCTTTGTTG- Bio-TEG

Probe 7	GAAGGGGCAGAGCAAGGAAA- Bio-TEG	
Probe 8	GAGGCAATCTCTTGGTTTTA- Bio-TEG	
**Antibodies**		
anti-LDB1	Abcam	Cat# ab96799; RRID:AB_10679400
anti-histone H3	Abcam	Cat# ab1791; RRID:AB_302613
anti-histone H3K27ac	Abcam	Cat# ab4729; RRID:AB_2118291
anti-Histone H3K9me3	Abcam	Cat# ab8898; RRID:AB_306848
anti-Tubulin	Abcam	Cat# ab7291; RRID:AB_2241126
anti-TBP	Abcam	Cat# ab63766; RRID:AB_1281140
anti-KLF1	Active Motif	Cat# 61233; RRID:AB_2615069
anti-SET1a	Bethyl Laboratories	Cat# A700-024-T; RRID:AB_2891825
anti-Pol II Ser2	Cell signaling	Cat# 13499; RRID:AB_11378081
anti-Pol II Ser5	Cell signaling	Cat# 13523; RRID:AB_11470388
anti-WDR5	Cell signaling	Cat# 13105; RRID:AB_2620133
anti-MLL1 N-Term	Cell signaling	Cat# 14689; RRID:AB_2688009
anti-MLL1 C-Term	Cell signaling	Cat# 14197; RRID:AB_2688010
anti-MENIN	Cell signaling	Cat# 6891; RRID:AB_10858216
anti-SET1b	Cell signaling	Cat# 44922; RRID:AB_2799275
anti-GAPDH	Cell signaling	Cat# 2118; RRID:AB_561053
anti-Pol II	Millipore Sigma	Cat# 05-623; RRID:AB_309852
anti-GATA1	Santa Cruz	Cat# sc-1233; RRID:AB_631559
anti-TAL1	Santa Cruz	Cat# sc12984; RRID:AB_2199699
anti-Pol II	Santa Cruz	Cat# sc-900; RRID:AB_2167474
Normal goat IgG	Santa Cruz	Cat# sc-2028; RRID:AB_737167
Normal mouse IgG	Santa Cruz	Cat# sc-2025; RRID:AB_737182

Normal rabbit IgG	Santa Cruz	Cat# sc-2027; RRID:AB_737197
