# Supplementary material for: An enhancer RNA recruits MLL1 to regulate transcription of Myb
Source: bioRxiv. 2023 Sep 26:2023.09.26.559528. Preprint. [Version 1] doi: 10.1101/2023.09.26.559528 (PMC10557664; doi:10.1101/2023.09.26.559528)
Supplement: Supplementary file 1 [file media-1.pdf]

SUPPLEMENTARY MATERIALS

CUT&Tag data analysis

Raw fastq pairs were preprocessed and removed adapter and low-quality sequences using the cutadapt program (v2.7). Preprocessed reads were aligned to the mouse genome available at Gencode M18 (Frankish, Carbonell-Sala *et al*., 2023) using Bowtie2 (v2.3.5) (Langmead and Salzberg, 2012) with the settings for Cut&Tag (Kaya-Okur, Wu *et al*., 2019). Final reads were retained after removing non-uniquely mapped reads using samtools (v1.9) with "-q 20" (Danecek, Bonfield *et al*., 2021) and duplicated reads using picard (v2.21.4) (http://broadinstitute.github.io/picard). Peaks were called using MACS2 (Zhang, Liu *et al*., 2008, Liu, 2014) (version 2.2.7.1). Browser track files were generated using the deepTools (Ramirez, Dundar *et al*., 2014) (v3.3.1).

RNA polymerase II (pol2) pausing index at *Myb* locus

For pol2 abundance over specific regions and pol2 pausing analysis, bam files were filtered for properly paired reads only with samtools view, and then converted to bedpe format using bedtools bamtobed. The subset of fragments that were shorter than 1000bp and mapped to chromosomes chr1-19,X,Y was extracted to generate a bed file of fragments. Subsets of bed files were generated based on the desired size ranges (total, <120bp, 120-270bp, >270bp). Fragments over genomic regions of interest were counted with bedtools intersect. The pol2 pausing index was calculated as the ratio of the fragments per million (FPM) in the first exon of *Myb* over the rest of the gene body.

Data availability

ENCODE data can be obtained from integrative publication PMID: 22955616; PMC: PMC3439153. Sequencing data are deposited in the Gene Expression Omnibus with accession code GSE240060.

REFERENCES

Danecek, P., J. K. Bonfield, J. Liddle, J. Marshall, V. Ohan, M. O. Pollard, A. Whitwham, T. Keane, S. A. McCarthy, R. M. Davies and H. Li (2021). "Twelve years of SAMtools and BCFtools." Gigascience 10(2).

Frankish, A., S. Carbonell-Sala, M. Diekhans, I. Jungreis, J. E. Loveland, J. M. Mudge, C. Sisu, J. C. Wright, C. Arnan, I. Barnes, A. Banerjee, R. Bennett, A. Berry, A. Bignell, C. Boix, F. Calvet, D. Cerdan-Velez, F. Cunningham, C. Davidson, S. Donaldson, C. Dursun, R. Fatima, S. Giorgetti, C. G. Giron, J. M. Gonzalez, M. Hardy, P. W. Harrison, T. Hourlier, Z. Hollis, T. Hunt, B. James, Y. Jiang, R. Johnson, M. Kay, J. Lagarde, F. J. Martin, L. M. Gomez, S. Nair, P. Ni, F. Pozo, V. Ramalingam, M. Ruffier, B. M. Schmitt, J. M. Schreiber, E. Steed, M. M. Suner, D. Sumathipala, I. Sycheva, B. Uszczynska-Ratajczak, E. Wass, Y. T. Yang, A. Yates, Z. Zafrulla, J. S. Choudhary, M. Gerstein, R. Guigo, T. J. P. Hubbard, M. Kellis, A. Kundaje, B. Paten, M. L. Tress and P. Flicek (2023). "GENCODE: reference annotation for the human and mouse genomes in 2023." Nucleic Acids Res 51(D1): D942-D949.

Kaya-Okur, H. S., S. J. Wu, C. A. Codomo, E. S. Pledger, T. D. Bryson, J. G. Henikoff, K. Ahmad and S. Henikoff (2019). "CUT&Tag for efficient epigenomic profiling of small samples and single cells." Nat Commun 10(1): 1930.

Langmead, B. and S. L. Salzberg (2012). "Fast gapped-read alignment with Bowtie 2." Nat Methods 9(4): 357-359.

Liu, T. (2014). "Use model-based Analysis of ChIP-Seq (MACS) to analyze short reads generated by sequencing protein-DNA interactions in embryonic stem cells." Methods Mol Biol 1150: 81-95.

Ramirez, F., F. Dundar, S. Diehl, B. A. Gruning and T. Manke (2014). "deepTools: a flexible platform for exploring deep-sequencing data." Nucleic Acids Res 42(Web Server issue): W187-191.

Zhang, Y., T. Liu, C. A. Meyer, J. Eeckhoute, D. S. Johnson, B. E. Bernstein, C. Nusbaum, R. M. Myers, M. Brown, W. Li and X. S. Liu (2008). "Model-based analysis of ChIP-Seq (MACS)." Genome Biol 9(9): R137.

Figure S1

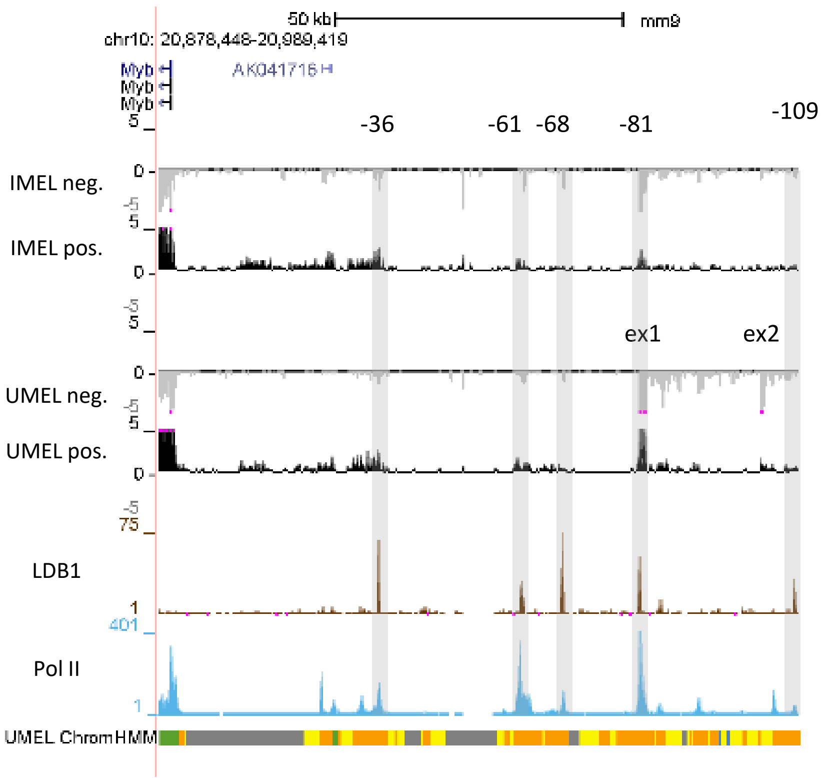

Figure S1. RNA-seq results for the *Myb-Hbsl1* intergenic region. Signal tracks for RNA-seq of induced (IMEL) and uninduced (UMEL) MEL cells from ENCODE/PSU are shown together with signal tracks for LDB1 and Pol II from ENCODE/Stan-Yale.

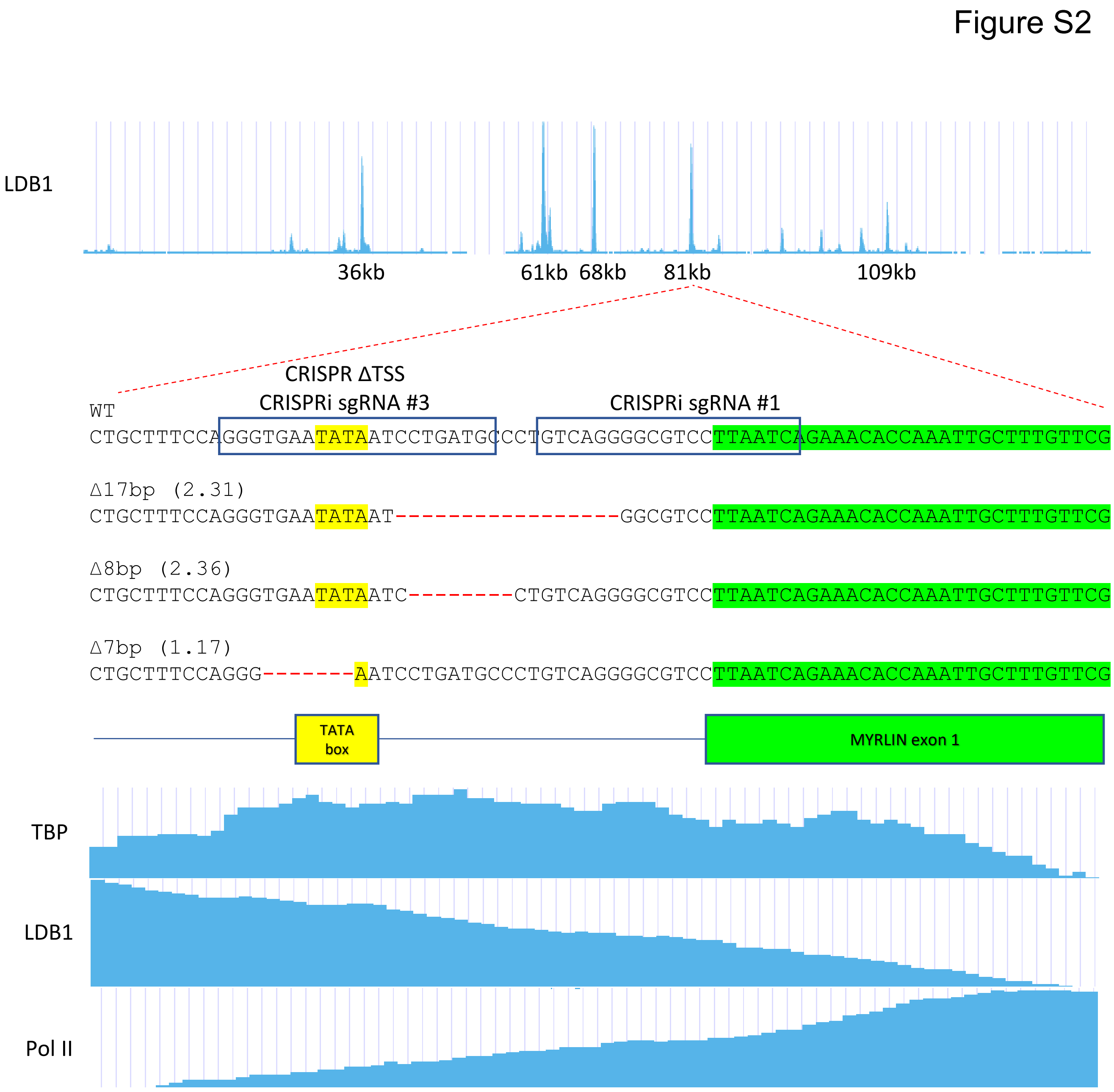

Figure S2. Schematic of the *Myb-Hbsl1* intergenic region. The zoomed in portion at the -81 kb enhancer shows the gRNA used for targeting the *Myrlin* TSS, the resulting three CRISPR-Cas9 deletions from Figure 2, the TATA box and the transcription start site for *Myrlin*. Below are signal tracks for TBP, LDB1 and Pol II from ENCODE/Stan-Yale. The locations of gRNAs used for CRISPR targeting of dCAS9-KRAB are also indicated.

Figure S3

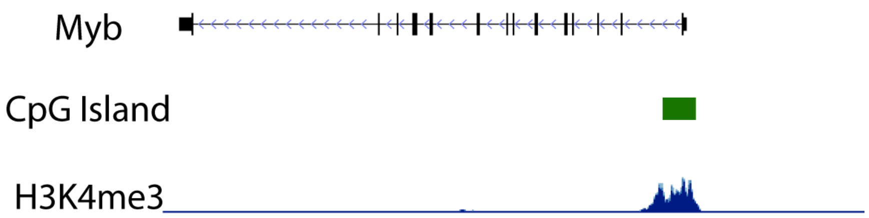

Figure S3. Encode genome browser view of the CpG island in the *Myb* promoter. The *Myb* promoter and first exon/intron contain a CpG island, which is highly enriched for H3K4me3 when *Myb* is actively transcribed.

Figure S4

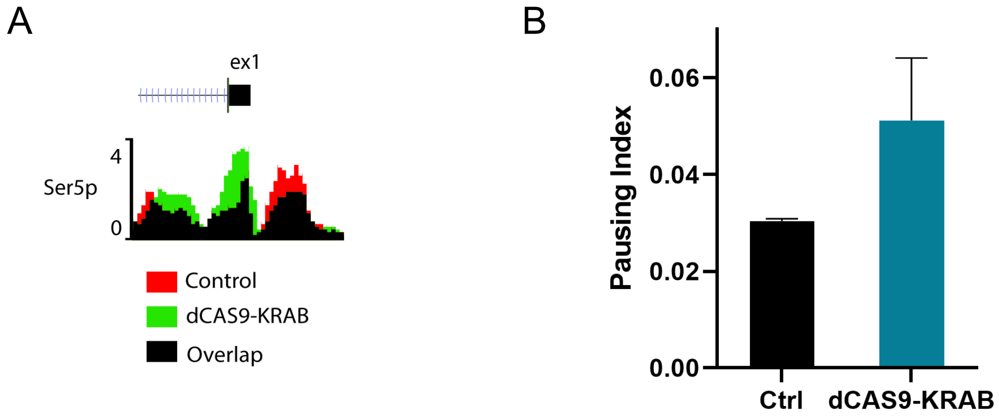

Figure S4. Pol II Ser5P occupancy in the *Myb* promoter. (A) Analysis of the Pol II Ser5P CUT & TAG fragments displaying Pol II Ser5 occupancy. (B) Pausing index calculated for Pol II Ser5 across *Myb*. Error bars indicate SEM of 3 independent experiments.

Figure S5

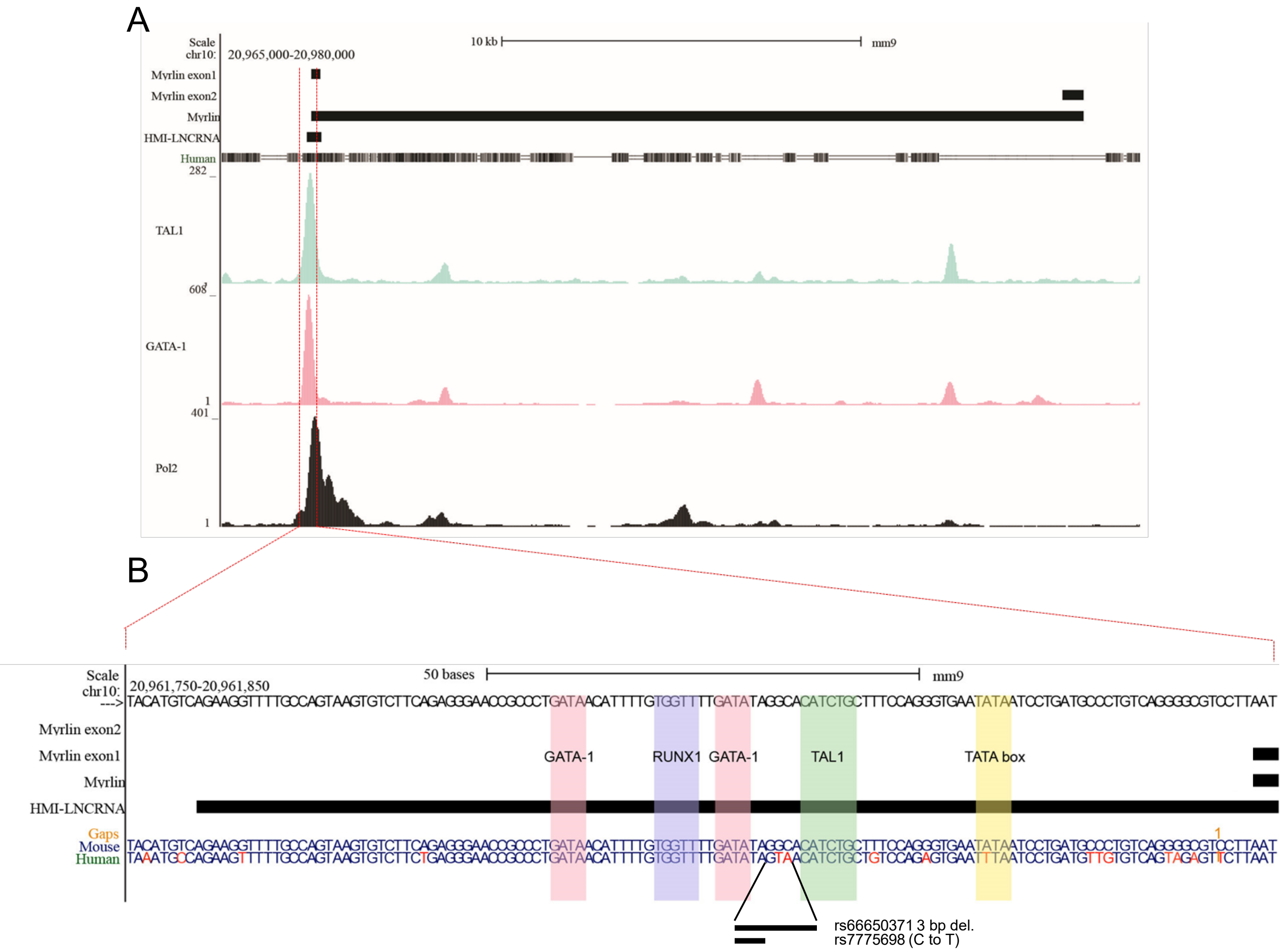

Figure S5. Human and mouse *Myb* enhancer transcripts. (A) Illustration of genomic region between chr10: 20,965,000 – 20,980,000 (mm9), showing BLATN result for human HMI-LNCRNA lncRNA and a 378 bp homology patch (see text) shared with the mouse *Myrlin* locus. Below are ChIP-seq signal tracks for TAL1, GATA1, and Pol2 from ENCODE/Stan-Yale at the -81 kb intergenic enhancer. (B) Chr10: 20,961,750 – 20,961,850 (mm9) zoom-in snapshot (dashed red lines) including HMI-LNCRNA DNA homology patch and highlighting GATA1, RUNX1, and TAL1 motifs conserved between mouse and human. The TATA box motif (yellow) appears only in the mouse reference genome. Below are the mouse and human DNA sequence showing nucleotide differences (red) with human single nucleotide polymorphisms (SNPs) rs66650371 and rs7775698 that are among the SNPs in the *MYB* locus that are linked to elevated fetal hemoglobin in humans (Farrell et al., 2011; Wang et al., 2018).

Table S1: Primers and sgRNAs

3' RACE outer	GAGTTTCACAACCACCTTCCAA
3' RACE inner	GAGAACAACAGTGGATGGGC
5' RACE outer	TAGCTGAGGGAACAAACGGAG
5' RACE inner	CTCTGGCATAACTGGAGTAAATTA
**PCR**	
Myrlin exon 1 F	TTAATCAGAAACACCAAATTGCTT
Myrlin exon 1 F	CTGTAGCTGAGGGAACAAACG
Myrlin exon 2 F	ATCAGGATACAGCTCTCAGCTACT
Myrlin exon 2 R	CGAGGCAATCTCTTGGTTTTA
**Real Time & ChIP qPCR**	
Actin exon F	ACCTTCTACAATGAGCTGCG
Actin exon R	CTGGATGGCTACGTACATGG
Actin intron F	GGGGCTCCACTTAGACCTAC
Actin intron R	GGCTCGTGTGACAAAGCTAA
Myrlin exon 1 F	AAATTGCTTTGTTCGCCTGC
Myrlin exon 1 R	CGAGTACCGCTTTCACAGTT
Myrlin exon 1/2 junction F	GTTCCGGCTCCGTTTGTT
Myrlin exon 1/2 junction R	CATCATTATGGTGGGGAACAT
Myrlin exon 2 F	TCAGCTACTGTTCCAGTGCC
Myrlin exon 2 R	GGGCTTACAGTTCCAGAGGA
Malat1 F	CAGGAGTGAGGCTTGTGGTA
Malat1 R	TGAATGGTAGAGCCAGCACA
Myb prom F	TCTTTGTTTGATGGCATCTGTT
Myb prom R	AAAGGGGAGGAGAAGGAGGT
-36kb F	TCACTTCCTTCCTGTCTCG
-36kb R	GTCTGGTGGCGATGACTTA
-61kb F	GTTGGGCAAAGATACTGGAT
-61kb R	GTTGGGCAAAGATACTGGAT
-68kb F	ATCCAAGTGACGGTGACA
-68kb R	GCATCCTGATTGTGCTAACT
-81kb F	CCCAAGTGAGAGAAATGTTGAA
-81kb R	TGTTATCAGGGCGGTTCC
-109kb F	CTCCAACATCAGCCGACT
-109kb R	ATTGTGAGGTGAGTGCCC
Myb exon 2 F	TTTCCAGATTTGGGCAGCAG
Myb exon 2 R	TGCAGCATCTACAGTAGCGA
Myb exon 3 F	GGTCCAGGGACCTTTGATGA
Myb exon 3 R	GACAGATGTGCAGTGCCAA

Necdin F	AGC TCA TGT GGT ACG TGT TGG T
Necdin R	GCT GCC CAT GAC CTC TTT CA
HS2 F	ATG TTA GTG TCA GCA TAT TAC CGA TGT
HS2 R	AAT GCA GAG GCT CGT TAA TGT G
Myb int1 F	GAACTGCAAACCAGCCTCTT
Myb int1 R	GGGATGTTTCATCCTGAGAGC
Hbb-bh1 F	CCTGGCCATCATGGGAAAC
Hbb-bh1 R	CCCCAAGCCCAAGGATGT
Hbb-b1 F	CACATGCAGCTTGTCACAGT
Hbb-b1 R	GTCAAAGCCCATGGCAAGAA
Hbb-y F	GCTTGTCACAGTGCAGTTCA
Hbb-y R	CGATGGCCTGAATCACTTGG
**CRISPR-gRNA**	
gRNA1 -81kb TSS SE	CACCG
gRNA1 -81kb TSS AE	AAAC**TGATTAAGGACGCCCCTGAC**
gRNA2 -81kb TSS SE	CACCG**GACGCCCCTGACAGGGCATC**
gRNA2 -81kb TSS AE	AAAC**GATGCCCTGTCAGGGGCGTCC**
gRNA3 -81kb TSS SE	CACCG**CATCAGGATTATATTCACCC**
gRNA3 -81kb TSS AE	AAAC**GGGTGAATATAATCCTGATGC**
**Chromatin Capture 3C Taqman qPCR**	
**HindIII fragment**	
Myb prom universal F	AGTTCAAGACTTGTGCTGACTG
Myb prom universal Probe	5'-FAM-AACCCCTAAAGCACTTGGGACACTCACAC-3'-BHQ1
ERCC3 universal Probe	5'-FAM-TGTCTGGAGGCTTACTGCGCCTGGTCTA-3'-BHQ1
20898686 R	CCTAGGTTCTCCTCTCCTACCA
20904395 R	GACAATTTGACATGAATTGCAAGC
20909424 R	CAAGAACCAAGACGCCTCAG
20915439 R	AGTAAATCTTGCTGCCCTCAAG
20923231 R	TGCCTTGGGCAGTTCTATAGAG
20939397 R	TCCTTACTTCTGCTCTCAAACAC
20944478 R	CTTTGTAGGTCACTTTCTCCAGC
20947418 R	AGCAGGCTATTGTGAAAAGAGG
20950525 R	ACACATAGGCACAGAGGAAAGTT
20957119 R	GCAACCTTTTCACTGGCAGAAAT
20974886 R	GAAGACCACTTAGGTAAACACTTTG
20978637 R	TGACACATTTGCTGCGAACAG
20986398 R	TGGAAAAGATCAGTAAGGCCAGA
21006046 R	ACTACATAACTTGGGTGTGTTGG
21013311 R	TGCTTCTTGGTCCCGGTGT

Table S2: Antibodies

Name	Company	Catalog
Anti-GATA1 (C20)	Santa Cruz	sc-1233
Anti-CLIM2 (N18)	Santa Cruz	sc11198
Anti-LDB1	Abcam	ab96799
Anti-TAL1 (C21)	Santa Cruz	sc12984
Anti-Pol II (C21)	Santa Cruz	sc-900
Anti-Pol II (CTD4H8)	Millipore Sigma	05-623
Anti-Pol II Ser2 (E1Z3G)	Cell signaling	13499
Anti-Pol II Ser5 (D9N5I)	Cell signaling	13523
Anti-TATA binding protein (TBP)	Abcam	ab63766
Anti-histone H3	Abcam	ab1791
Anti-histone H3 (acetyl K27)	Abcam	ab4729
Anti-Histone H3 (tri methyl K9)	Abcam	ab8898
Anti-WDR5 (D9E1I)	Cell signaling	13105
Anti-MLL1 (D2M7U)	Cell signaling	14689
Anti-MLL1 (D6G8N)	Cell signaling	14197
Anti-MENIN (D45B1)	Cell signaling	6891
Anti-SET1a (D3V9S)	Bethyl Laboratories	A700-024-T
Anti-SET1b (D1U5D)	Cell signaling	44922
Anti-KLF1	Active Motif	61233
Anti-Tubulin [DM1A]	Abcam	AB7291
Normal goat IgG	Santa Cruz	sc-2028
Normal rabbit IgG	Santa Cruz	sc-2027
Normal mouse IgG	Santa Cruz	sc-2025
